# Barriers and enablers to promoting grandchildren’s physical activity and reducing screen time: a qualitative study with Australian grandparents

**DOI:** 10.1186/s12889-024-19178-2

**Published:** 2024-06-22

**Authors:** Timothy Budden, David A Coall, Ben Jackson, Hayley Christian, Andrea Nathan, Michelle I Jongenelis

**Affiliations:** 1 School of Human Sciences (Exercise and Sport Science), The University of Western Australia, 35 Stirling Highway, Nedlands, WA 6009 Australia; 2 Telethon Kids Institute, The University of Western Australia, 15 Hospital Avenue, Nedlands, WA 6009 Australia; 3 School of Medical and Health Sciences, Edith Cowan University, 270 Joondalup Drive, Joondalup, WA 6027 Australia; 4 School of Population and Global Health, The University of Western Australia, 35 Stirling Highway, Nedlands, WA 6009 Australia; 5 Melbourne Centre for Behaviour Change, Melbourne School of Psychological Sciences, The University of Melbourne, Grattan St, Melbourne, VIC 3010 Australia

**Keywords:** Grandparents, Grandchildren, Physical activity, Screen time, Strategies, Barriers, Enablers

## Abstract

**Background:**

With an increasing number of grandparents providing care to their grandchildren, calls have been made for these caregivers to be considered important stakeholders in encouraging children’s engagement in health-promoting behaviors, such as physical activity. Understanding the perspectives of grandparents who provide care is crucial to informing efforts that aim to increase children’s physical activity, yet little is understood about their perceptions of specific barriers and enablers to promoting children’s physical activity and reducing screen time. The present study sought to explore these perceptions.

**Methods:**

Semi-structured focus groups and individual interviews were conducted with grandparents who reported providing care to a grandchild aged 3 to 14 years. A total of 20 grandparents were sampled (mean age = 67.8 years). Data were subjected to reflexive thematic analysis.

**Results:**

Key reported barriers to physical activity included (i) the effort (physical and logistical) and financial cost associated with organizing physical activities, (ii) grandparents’ age and mobility issues (e.g., due to injury or illness), (iii) caring for children of different ages (e.g., older children having different physical activity interests than younger children), and (iv) a local environment that is not conducive to physical activity (e.g., lack of appropriate facilities). Barriers to reducing screen time included (i) parents sending children to care with electronic devices and (ii) children’s fear of missing out on social connection that occurs electronically. Strategies and enablers of physical activity included (i) integrating activity into caregiving routines (e.g., walking the dog), (ii) involving grandchildren in decision making (e.g., asking them in which physical activities they wish to engage), (iii) encouraging grandchildren to engage in activity with other children, and (iv) creating a physical and social environment that supports activity (e.g., owning play equipment). A common strategy for reducing screen time was the creation of a home environment that is not conducive to this activity (e.g., removing electronic devices from view).

**Conclusions:**

Findings suggest that grandparents may benefit from resources that assist them to identify activities that are inexpensive and require minimal effort to organize. Activities that account for grandparents’ age and health status, as well as any environmental barriers, are likely to be well-received.

## Introduction

Physical inactivity and sedentary behavior are modifiable risk factors that play a critical role in the development of multiple non-communicable diseases, including cardiovascular disease, stroke, hypertension, and osteoporosis [1–5]. By contrast, regular engagement in physical activity reduces morbidity and all-cause mortality and improves mental health outcomes [6–9]. Physical activity patterns are established early in life and persist into adulthood [10, 11], highlighting the importance of promoting active lifestyles in childhood. Despite this, a majority of Australian children do not meet the recommendations outlined in the 24-hour movement guidelines [12, 13]. Improving children’s movement behaviors (e.g., increasing physical activity, reducing sedentary behavior) has thus become an important part of public health agendas [14, 15].

The family environment plays an influential role in shaping children’s health habits. The importance of parents in promoting physical activity in children is particularly well-documented, with parents influencing their children’s physical activity habits by educating on, supporting, and modelling movement behaviors [16–20]. With an increasing number of grandparents providing childcare [21, 22], calls have been made for these caregivers to be considered important stakeholders in encouraging children’s health-promoting behaviors [23–25], including physical activity [26]. Grandparents and grandchildren play important social roles in each others’ lives, with these roles often extending beyond frequency of contact and relationship closeness [27]. Research exploring the role of grandparents in their grandchildren’s movement behaviors is beginning to emerge, with findings to date suggesting that children’s engagement in physical activity reflects the activity levels of their caregivers, including grandparents, and that grandparents may be more permissive about screen-based activity compared to parents [28, 29]. Other work suggests there is considerable opportunity to improve children’s movement behaviors while they are in grandparental care, with high levels of engagement in screen-based activities identified [30].

An understanding of grandparents’ perceptions of the barriers to supporting their grandchildren’s movement behaviors is critical to informing efforts to improve children’s physical activity levels. However, research in this space is limited. In a sample of Latino grandparents, reported barriers to promoting grandchildren’s physical activity included lack of information, transport, money, time, and access to affordable and safe physical activity facilities or programs [31]. More recently, Parrish et al. [32] explored the role of parents and grandparents in supporting children’s physical activity. Although this study did not explicitly examine barriers or enablers of physical activity, results suggested that (i) the appropriateness (e.g., safety) of outdoor versus indoor spaces for physical activity, (ii) the presence (or absence) of active spaces, and (iii) caregiver availability and energy may be important considerations. Specific barriers and enablers to reducing grandchildren’s screen time were not explored in either study.

Research that seeks to understand the perspectives of grandparents is critical to improving knowledge of the grandparent-related drivers of movement behaviors among children. Given limited prior work, especially in relation to screen time, the present study sought to explore grandparents’ perceptions of the (i) barriers to promoting their grandchildren’s physical activity and reducing screen time and (ii) enablers of physical activity and reduced screen time.

## Methods

### Participants

A market research agency was commissioned to recruit a sample of Australian grandparents. To be eligible for this study, grandparents must have been providing regular care (defined as ≥ 3 h per week) to a grandchild aged 3 to 14 years. To account for the lived experiences of those from low socioeconomic and regional areas, participants were recruited from low, middle, and high socioeconomic status (SES) neighborhoods, and from metropolitan and regional locations in Western Australia. Participants (*n* = 20; 16 women, 4 men) ranged in age from 59 to 82 years (*M* = 67.80, *SD* = 5.33).

As prior research has found that 80% of all focus group themes are discoverable within 2–3 focus groups and 90% are discoverable within 3–6, four focus groups were scheduled [33]. Due to low attendance at one of these focus groups, two individual interviews were conducted instead, and the data from these interviews were combined with the data from the three focus groups [34]. The profile of each group can be found in Table 1.

**Table 1:** Focus group composition

Group	*N*	Gender	Location	Socio-economic status^a^
1	5	Mixed (2 men, 3 women)	Regional	Low-Mid
2	6	Women	Metropolitan	Low
3	7	Women	Metropolitan	Mid-High
Individual	2	Men	Metropolitan	Mid-High

^a^Based on the Index of Relative Socio-economic Advantage and Disadvantage [56]

### Procedures

Ethics approval to conduct the study was received from The University of Melbourne. All participants provided written informed consent prior to their participation. Participants were reimbursed AUD90 for their time and costs associated with focus group or interview attendance. Focus groups were conducted at local community centres or health service agencies and were approximately 90 min in duration. Individual interviews were also conducted at these locations and ranged from 40 to 60 min in duration. Data were collected in October-December 2022.

Participants completed a brief survey while waiting for their focus group or interview to begin. Items in the survey were designed to gather descriptive information about the sample and thus assessed participants’ demographic characteristics (gender, age, postcode, and level of education), as well as their engagement in physical activity and screen time. Participants were also asked to report on their grandchildren’s engagement in physical activity and screen time while in grandparental care. Focus groups then started with introductions. A semi-structured interview guide comprising open-ended questions was followed. This facilitated dialogue between participants and allowed the researcher to comprehensively explore grandparents’ perceptions of the barriers and enablers to supporting their grandchildren’s engagement in physical activity and reducing screen time while in their care. Responses to the following questions were of primary interest:

1. What gets in the way of your grandkids being physically active when you are looking after them?
2. What do you find helps getting your grandkids physically active?
3. What gets in the way of reducing your grandkids’ screen time?
4. What do you find helps getting your grandkids off screens?

Probing questions were used to generate further information on specific topics of interest, and points raised by participants were written on a whiteboard to prompt further discussion and synthesize information.

Focus groups were facilitated by MJ, a principal research fellow with a PhD in clinical psychology. Support was provided by TB, an early career researcher with experience conducting qualitative research with a variety of populations. MJ and TB each conducted one of the individual interviews. Focus groups and interviews were audio-recorded and then transcribed verbatim by an ISO-accredited transcription service. Transcripts were imported into NVivo for coding and analysis.

### Data analysis

We adopted an interpretivist paradigm in this study, underpinned by ontological relativism and a subjectivist epistemology [35]. In line with the assumptions underpinning this approach, we conducted reflexive thematic analysis and adopted the six-step thematic analysis procedure outlined by Braun and Clarke [36–38]. A semantic approach to thematic analysis was adopted. Consistent with this approach, we assumed that participants’ reports were accurate and trustworthy representations of their thoughts and behaviors [37]. Such an approach is appropriate when the aim is to generate novel insights into a phenomenon or issue important to participants [39].

Coding and analysis were conducted by the lead author, TB, a 27-year-old male from an English-speaking background. TB was significantly younger than the participants and had no personal experience with grandparents regarding physical activity or sedentary behaviour. Although this lack of shared experience with the participants may have limited common ground, it provided an opportunity to garner fresh insights as content that might not seem novel or unique to someone with relevant personal experiences could appear more striking.

Initially, TB familiarized himself with the data by reading transcripts in their entirety and generating initial codes of the dataset. TB also transposed the information written on the whiteboards into NVivo and cross-referenced this information with the transcripts to ensure the content closely matched the discussions. Following this, in a series of ‘critical friends’ meetings, TB and MJ reviewed and refined the codes identified in the analysis process, and generated key themes based on these codes.

Quotes are provided throughout the results section to highlight the barriers and enablers reported by grandparents. Each participant quote is followed by details of the focus group (e.g., FG1, FG2, or FG3) or individual interview (e.g., P1 or P2) of which the participant was a part. The guidelines outlined in the Standards for Reporting Qualitative Research were used to prepare this manuscript [40] .

## Results

### Descriptive information

All but one participant provided secondary care to a grandchild aged 3 to 14 years. Participants provided an average of 25.27 h of care per week (*SD* = 37.42). Excluding the one participant providing primary care (i.e., reported providing 24/7 care), the average amount of care provided per week was 17.76 h (range 2–72 h, *SD* = 16.94). Nearly two-thirds (60%) of the sample reported engaging in moderate or vigorous work-related activities; 83% reported engaging in moderate or vigorous sport, fitness, and recreational activities; and 55% reported engaging in active travel. Participants reported spending 12.18 h per week watching television, 1.55 h per week playing video games, and 7.25 h per week on a computer or smartphone.

### Barriers to increasing grandchildren’s physical activity and reducing screen time

Grandparents reported several barriers to increasing their grandchildren’s physical activity (Table 2) and reducing their screen time (Table 3). These barriers spanned the levels of ‘grandparent(s)’, ‘grandchild(ren)’, ‘parent(s) of grandchild(ren)’, and the ‘local environment’. Barriers are presented below in order of relative importance; defined as the frequency with which participants reported experiencing the barrier and the number of participants who reported the barrier.

**Table 2:** Grandparents’ reports of the barriers to promoting their grandchildren’s physical activity

Barrier	Category	Description
Effort and financial cost	Grandparent(s)	The burden associated with organizing grandchildren’s physical activities, including preparing food, clothes, and paying for costly trips.
Ageing: poor health and injury	Grandparent(s)	Issues related to ageing, including poor health and injury, that impact grandparents’ ability to engage in physical activity with their grandchildren.
Age (differences) of grandchildren	Grandchild(ren)	Difficulties promoting physical activity when caring for children of different ages. Difficulties caring for older children due to their access to, and experience with, screens and electronic devices.
Grandparent home environment	Grandparent(s)	The home environment is not conducive to physical activity (e.g., a small apartment) and is filled with technological distractions (i.e., screens).
Lack of safety	Local environment	The local neighborhood has few areas perceived as safe for grandchildren to engage in physical activity.
Lack of facilities	Local environment	The local neighborhood has few facilities suitable for grandchildren’s physical activity (often due to closures resulting from COVID-19).
Weather	Local environment	Weather can prohibit or make engagement in physical activity unattractive (e.g., too hot, heavy rain, storms).
Fear of injury/illness	Grandchild(ren)	Children are afraid of getting injured or contracting viruses (particularly during the COVID-19 pandemic), contributing to anxiety and avoidance of physical activity.
Parental attitudes	Parent(s) of grandchild(ren)	Some parent(s) have negative attitudes towards grandparents’ involvement in physical activity, leading to conflict and limiting grandparents’ ability to promote grandchildren’s physical activity.
Differing household dynamics	Parent(s) of grandchild(ren)	Some parent(s)’ rules and household dynamics regarding physical activity participation (and screen time use) conflict with grandparents’ rules and undermine ability to promote physical activity.

**Table 3:** Grandparents’ reports of the barriers to reducing grandchildren’s screen time

Barrier	Category	Description
Parents send grandchildren with devices	Parent(s) of grandchild(ren)	Parents often use screens as ‘babysitters’ and frequently send children to their grandparents with a device. This often conflicts with grandparents’ desire to have a ‘device free’ home.
Fear of missing out	Grandchild(ren)	Children fear missing out on social connection with peers if they do not have their devices.
Age of grandchildren	Grandchild(ren)	Greater difficulties reducing older grandchildren’s screen use than younger grandchildren’s.
Grandparents need a break during care	Grandparent(s)	Some grandparents report using devices to provide themselves with a break/respite when caring for grandchildren.
Strategies employed by grandchildren	Grandchild(ren)	Children occasionally use strategies, such as ‘divide and conquer’, to mislead grandparents regarding the rules their parents set for screen use.
Grandchildren have ‘inactive’ personalities	Grandchild(ren)	Personalities of grandchildren differ substantially, particularly in how responsive they are to suggestions to replace screen use with physical activity.
Health of grandchild(ren)	Grandchild(ren)	Children with health conditions (e.g., ADHD, anxiety) may require access to devices or are encouraged to use certain applications on devices.

One of the primary barriers to promoting physical activity was the substantial effort involved in its organization, with participants citing the burden associated with (i) planning suitable activities, (ii) preparing food, clothing, and transport for those activities, and (ii) the financial cost of some activities. A trip to the beach, for example, was an effortful task, as described by this participant (FG1, Mixed, Regional, Low-Mid SES):

*…so you think, oh, rethink. Who can I take with me next time? But when you pack up and you’re tired, then you’ve got wet towels, they’re dragging them along the ground, they’ve got toys they wanted to bring and it’s just doing a head count and getting everything back into the boot of the car, doing up four seatbelts and things like that….*

Other factors that participants believed made promoting physical activity difficult included their age and health/injury issues, and the age of the grandchildren for whom they provide care. In terms of their age and health/injury issues, grandparents reported that these affected their ability to participate in physical activity with their grandchildren. Specifically, grandparents noted that their “*limited capacity*”, “*aches*”, and “*pains*” – and that they were not “*quite as fast*” as they were when they were younger and healthier – made it a “*challenge to keep up*” with (multiple) energetic young grandchildren. In terms of the age of the grandchildren for whom they provide care, participants noted that as grandchildren enter late childhood/early adolescence, their interests change and they have increased access to, and experience with, screens and electronic devices. Providing care for multiple grandchildren, especially those of different ages, was considered a challenge due to their varied desires and interests and the associated difficulties arranging activities that would keep all grandchildren engaged:

*I find another barrier too [is], with my lot, that the age difference is difficult…because some of them want to go to the park or they want to do something and…someone else wants to do [something else] and we have to all go and do one thing, we can’t separate.* (FG3, Women, Metropolitan, Mid-High SES)

A home environment that was not conducive to physical activity and perceived issues with the local neighborhood were also cited as barriers. In terms of the former, grandparents living in apartments reported finding it difficult to promote physical activity in such a small space while others commented on the multiple electronic devices in their home and the difficulties moderating screen time as a result. In terms of the local neighborhood, grandparents were often concerned about safety and their ability to protect their grandchildren from harm. Some neighborhoods were considered to be lacking attractive or viable facilities for physical activity (often due to closures arising from the COVID-19 pandemic).

*Yeah, if you go to some of the parks that aren’t fenced and if you’ve got a little one, a two-year-old who can run really fast and you can’t run really fast…* (FG1, Mixed, Regional, Low-Mid SES).

The attitudes and behaviors of the parents of the children for whom participants provided care were considered barriers to both promoting physical activity and reducing screen time. Participants noted that some parents are supportive of children’s use of electronic devices and less concerned with their children’s physical activity, resulting in a different set of rules in the family home compared to those in the grandparental home. Grandparents lamented that devices are sometimes used by parents as “*babysitters*”, although some grandparents noted that they also resorted to using devices when needing a break:

*Well, I must say, I do that. By about six o’clock at night I’ve had enough, I’m exhausted and the shift’s still going, so I’ve said, “watch Bluey” [an Australian TV show].* (FG3, Women, Metropolitan, Mid-High SES)

Relatedly, participants reported that children often came to their home with the expectation that the same rules as those in the parents’ household applied. In some instances, grandparents struggled to enforce their own household rules when parents sent children to them with electronic devices in hand:

*My two they always come with their iPads, and we don’t like it. Just keep on telling the parents not to bring the kids with those iPads. But they still come.* (FG2, Women, Metropolitan, Low SES)

According to some grandparents, children were adept at “*pushing the boundaries*” and challenging their grandparents’ rules, which was a barrier to reducing screen time. Children reportedly engaged in strategies to “*divide and conquer*” parents and grandparents. For example, it was noted that some grandchildren attempt to convince their grandparents to provide them with access to electronic devices by giving misleading information about the rules regarding screen time that are present in the family home:

*The way they play you off with their parents – “I’m allowed to do that at home” – whether they are or not.* (FG3, Women, Metropolitan, Mid-High SES)

In terms of the barriers to minimizing screen time specifically, grandparents spoke of some grandchildren having “*inactive personalities*”: they tended to avoid physical activity and opted instead for more sedentary activities, including (but not limited to) screen-based activities. They also reported that for many grandchildren, especially those who were older, electronic devices were a means by which they connected with peers and they feared missing out on this social connection. Finally, screen time was considered important for managing children’s ADHD or anxiety, with some grandparents reporting that the family doctor encouraged the use of various apps, or devices in general, as a means of managing behavioral issues:

*[The pediatrician] told my daughter, because we were restricting [screen use], [my daughter] was restricting it and everything and taking it off him, and [the pediatrician] said “Do not restrict him with that”. No, really, because he would break a mirror, break glass.* (FG2, Women, Metropolitan, Low SES)

### Strategies and enablers for increasing physical activity and reducing screen time

#### Strategies and enablers for increasing physical activity

The strategies and enablers for increasing physical activity reported by participants tended to complement the aforementioned barriers (see Table 4). Strategies included (i) integrating physical activity into daily routines, (ii) involving grandchildren in decisions relating to physical activity, (iii) encouraging grandchildren to participate in physical activity with each other and with other children, and (iv) creating an environment that supports physical activity engagement. Enablers included (i) residing in a neighborhood with safe and well-equipped outdoor areas (such as parks and playgrounds) and indoor facilities (such as swimming pools) and (ii) parents and grandparents “*being on the same page*” about physical activity.

**Table 4:** Strategies and enablers for promoting grandchildren’s physical activity

Strategies / Enablers	Description
Integrating physical activity into daily routines	Reducing the amount of effort required to promote physical activity by incorporating activity into their routine with grandchildren (e.g., by walking the dog, walking to school, or involving grandchildren in household chores).
Involving grandchildren in decision making	Allowing grandchildren to make decisions regarding physical activity (e.g., selecting and planning activities), providing grandchildren with autonomy, and listening to grandchildren.
Encouraging grandchildren to engage in physical activity together	Grandchildren can teach each other how to engage in physical activity (e.g., by learning new skills, such as skateboarding) and engage in physical activity together.
Creating a home environment that supports physical activity	Strategies to create an environment that supports physical activity, including having a variety of equipment in the home; requesting (and having) parents send children with equipment (e.g., bikes, sport-specific equipment); limiting screen time; and setting rules regarding screen use in the grandparental home.
Suitable local environment for physical activity	Local neighborhood has a variety of suitable (and safe) locations, such as cul-de-sacs, well-equipped playgrounds, and parks, that allow grandchildren to engage in physical activity if they are unable to do so at the grandparents’ home.
High-quality public facilities	Local neighborhood has affordable and high-quality public indoor facilities (e.g., swimming pools, community classes for children).
Parents and grandparents are “on the same page” and supportive of physical activity	Grandparents and parents discuss physical activity and parents support grandparents’ involvement in their grandchildren’s physical activity (e.g., by providing financial resources or sending children to their grandparents without devices).

To reduce the effort involved in promoting children’s physical activity, grandparents suggested integrating physical activity into daily routines. Specific suggestions included walking the dog, walking children to school, or involving grandchildren in household chores.

*Yeah, I’ve got a dog and my grandkids don’t have a dog…so every time they come to my place it’s take the dog for a walk. We go for a walk down to the park with a ball and they just like doing that.* (FG3, Women, Metropolitan, Mid-High SES)

Providing care for multiple grandchildren of the same age at once was also believed to reduce some of the effort grandparents needed to engage grandchildren in physical activity, with children serving as role models for each other:

*It helps when there’s a few of them. See I’ve only got one now that I look after but I’ve looked after them in groups before and they do, as you said before, they encourage each other. They’re never inside, always outside. It’s a bit more difficult when I’ve just got one.* (FG2, Women, Metropolitan, Low SES)

When selecting physical activities for children, several grandparents discussed the importance of involving grandchildren in decision making and, by extension, providing children with autonomy. Participants varied in the extent to which they involved grandchildren in decisions around physical activity. Some noted that grandchildren only have a “*say in the plan*” that the grandparent already developed for the child’s visit. Others involved grandchildren to a greater extent, particularly during the initial planning stages, by asking their grandchildren what they felt like doing and developing a plan based on their response. Some grandparents reported that their grandchildren arrived for care with detailed plans, or an “*itinerary*” from their parents.

Participants described the importance of creating a home environment that supports physical activity. Owning a variety of play equipment (e.g., bikes, balls) was commonly reported. The local physical environment was also considered an important enabler of physical activity, particularly when the grandparents’ home was not suitable. Living near parks, skate-parks, rivers, local obstacle courses, and other quality public facilities, or living in cul-de-sacs with other families nearby, were thought to facilitate outdoor activity. Some grandparents reported actively searching for stimulating outdoor areas where their grandchildren could play, especially when their own physical limitations prevented them from engaging in physical activity with their grandchildren:

*I can’t get out and kick a football around with them or play cricket with them. So, I’m trying, I look for things like the treetop walks down at [south-western region of Western Australia] or go down to … to the obstacle courses and they go to swimming lessons.* (FG3, Women, Metropolitan, Mid-High SES)

Finally, grandparents described the importance of being “*on the same page*” as parents regarding physical activity. For example, P2 (Male, Metropolitan, Mid-High SES) reported sharing the view with his son that being physically active is important, and this led to mutual efforts to coordinate P2’s grandchild’s involvement in physical activities.

#### Strategies and enablers for reducing screen time

The strategies and enablers for reducing screen time reported by focus group participants are presented in Table 5. Creating a home environment that limits screen time generated much discussion. Strategies described by participants included having clear rules around device use, such as setting time limits on use. This often required a clear understanding between all parties that the grandparents’ home was different to the parents’ home; an understanding that was used by grandparents when their grandchildren pointed out that their parents allowed screen time. These boundaries were believed to assist with parents also, with some grandparents reporting that parents reinforced the rules when visiting the grandparents’ home:

*I’ve got an agreement with my [grand]kids, with their parents and my house, my rules. Even if they’re there it’s still my house, my rules and no one else has any other say.* (FG3, Women, Metropolitan, Mid-High SES)

**Table 5:** Strategies and enablers for reducing grandchildren’s screen time

Strategies / Enablers	Description
Creating a home environment that limits screen time	Adopting several strategies to create a home environment that limits grandchildren’s screen time, including (i) setting rules around screen time (e.g., time limits); (ii) setting rules regarding the presence of devices in the grandparents’ home; and (iii) unplugging the internet during grandchildren’s visits.
Distracting grandchildren with an attractive alternative to screens	Encouraging grandchildren to engage in physical activity or play board games. Providing grandchildren with food (e.g., snacks).
Provide care for grandchildren in groups	Providing care for multiple grandchildren sometimes leads to them engaging in activities together.
Reward systems (or ‘bribing’)	Grandparents incentivize non-screen activities (e.g., physical activity, chores, cooking), sometimes by ‘bribing’ grandchildren (e.g., with ‘pocket-money’).

*Participant: I said “I don’t care what your mum said. [Laughs] I don’t care.”*

*Participant: …but then if their mum or dad hears, their mum will tell them “When you’re at Nanna’s place it’s what she says, not what you want to do.”* (FG3, Women, Metropolitan, Mid-High SES).

Some grandparents reported avoiding the issue of screen time entirely by setting strict rules regarding devices in the home and communicating these rules with the parent of their grandchild. In preparation for caring for grandchildren, some grandparents reported that they removed all devices from view and instructed parents not to send grandchildren with any devices of their own. Some grandparents described turning off the internet during their grandchildren’s visits and then using the lack of internet to their advantage by “*saying something like ‘the WiFi’s down’ or ‘it’s not working, WiFi’s down’*” (FG3, Women, Metropolitan, Mid-High SES).

Some grandparents employed strategies that involved “*distracting*” their grandchildren from their devices by providing them with alternative activities, such as physical activity, games, or food. Grandparents reported that alternatives to screens needed to be stimulating enough to engage grandchildren for extended periods of time and may be more effective when grandchildren are younger:

*…if you just want to play hide and seek or something like that – she’s only young. You’re probably dealing with grandkids who are much older, who are more mature. But she’s still fairly young and so she still likes playing young person’s things. So you just have to suggest something, like some game that will just get her involved…* (P2, Male, Metropolitan, Mid-High SES).

Finally, some grandparents reported using incentives to reduce screen time, which they typically described as “*bribing*”:

*…in the afternoon when we’re back but I just want to just get him off the Xbox, I do find that – so either switch it off or saying “okay if you don’t come off now there’s no takeaway tonight”.* (P1, Male, Metropolitan, Mid-High SES)

## Discussion

Reflecting the increasing role grandparents are playing as caregivers for their grandchildren, and their likely importance in shaping their grandchildren’s movement behaviors, the present study sought to explore grandparents’ perceptions of the barriers and enablers to promoting children’s physical activity and reducing screen time. Several barriers and enablers were identified, which are discussed in detail below. We present implications of the research findings, most notably the importance of (i) developing resources that empower grandparents to support their grandchildren’s movement behaviors, (ii) facilitating access to affordable and high-quality local facilities for physical activity, and (iii) encouraging collaboration and effective communication between parents and grandparents regarding physical activity and screen time.

Frequently reported barriers to increasing grandchildren’s physical activity included the effort and cost involved in organizing activities; grandparents’ age, illnesses, and injuries; the age of the grandchildren for whom grandparents provide care; challenges that arise when providing care to multiple grandchildren of different ages; and the quality, safety, and availability of locations for physical activity in the local neighborhood. Results relating to the health/energy levels of caregivers and concerns about the local environment are consistent with prior work conducted in the US [31, 32]. In terms of grandparent health, programs aiming to increase children’s engagement in physical activity via their grandparents should account for the age, illnesses, or injuries that may limit grandparents’ ability to model physical activity for their grandchildren or participate in physical activity with them. Identifying and promoting suitable alternative activities in which both grandparents and their grandchildren can engage, and ensuring these activities are not burdensome, is warranted. Emphasizing to grandparents that they can support physical activity via other means, for example, by championing engagement and discussing its importance, also constitutes a potential means by which grandparents can be encouraged to promote physical activity.

In terms of the local neighborhood, results support previous research highlighting the importance of the built environment and the presence of local facilities that are considered safe, appropriate, and affordable or free to use [41]. This is particularly critical for grandparents whose home environment is not conducive to physical activity (e.g., apartment residences) and/or who may be constrained financially. Supporting grandparents to identify high quality and affordable local facilities is needed, especially given participants in the present study considered suitable local environments and high-quality public facilities to be important enablers of their grandchildren’s physical activity. Local government policies that (i) prioritize the development and maintenance of attractive and safe neighborhoods and (ii) support investment in programs that encourage physical activity in grandchildren and their grandparents are also likely to assist [42].

Barriers relating to the age of the grandchildren for whom grandparents provide care and the challenges that arise when providing care to multiple grandchildren of different ages are unique findings and speak to the importance of ensuring programs supporting grandparents include resources that account for the various child age groups for whom grandparents may be providing care. Suggestions for age-appropriate activities and, importantly, activities in which children of different ages can engage may assist with addressing the difficulties faced by grandparents caring for grandchildren of different ages and/or older children who have greater experience with electronic devices.

Results highlight the importance of ensuring grandparents and the parents of the children for whom they provide care are aligned in their views regarding physical activity and screen time. Participants expressed significant frustration at parents sending their children to be cared for with electronic devices, noting that this made it harder for them to enforce rules around screen time. This is consistent with research in the nutrition space that found disagreements between family members about eating practices complicate efforts to promote healthy eating among children [29, 43, 44]. Participants believed that being on the same page as the parents of the children for whom they provide care and involving children in decision making were important enablers of physical activity. These findings provide support for the development of family-based, intergenerational programs that encourage conversations and collaboration between grandparents, parents, and children about physical activity and screen time, thus improving communication and assisting caregivers to minimize any conflict.

Participants discussed a variety of factors they believed enabled physical activity. These included integrating physical activity into existing routines, involving children in decision making, creating a home environment that is conducive to physical activity, identifying attractive alternatives to screen time, and identifying suitable neighborhood locations for activities. In terms of integrating physical activity into existing routines, encouraging grandparents to engage in active transport with their grandchildren or asking their grandchildren to assist with walking the dog has the potential to increase physical activity in both grandchildren and grandparents while being less burdensome on grandparents. In terms of children’s involvement in decision making, this has the potential to confer a degree of autonomy, which is linked with motivation for physical activity [45]. With respect to the home environment, encouraging parents to send their children to care with some portable play equipment that facilitates physical activity (e.g., balls, bats, scooters) may assist grandparents with creating an environment that promotes physical activity.

In terms of screen time, grandparents highlighted the importance of setting limits for device use. Research conducted in the context of healthy eating suggests structure-based practices such as limit setting are a positive behavior management approach [46, 47]. Setting limits rather than restricting or allowing free access to foods fosters autonomy and self-control over eating decisions and may facilitate the formation of healthy habits, laying the foundation for healthy eating in adolescence and adulthood [48]. Research exploring the relationship between limit setting and screen time is limited to work conducted with parents and children. This research has found that parents who are aware of recommendations for daily screen time limits and ‘always’ or ‘very often’ set limits on screen use tend to have children who rarely exceed recommended limits [49]. Other research suggests that parents who set limits collaboratively with their children may be more effective in managing their children’s screen time [50]. Work exploring these relationships in grandparent care providers is warranted.

The consequences of ‘bribing’ children to reduce screen-time behavior require further exploration. Providing alternatives to screen time is likely to be a more appropriate strategy that should be encouraged. Finally, given participants’ reports that children challenged their grandparents’ rules and engaged in strategies to “divide and conquer” parents and grandparents, these caregivers may benefit from assertiveness skills training and/or resources that assist them to manage persistent requests for electronic devices.

### Limitations and future directions

The present study had several limitations. First, due to low attendance, we were only able to conduct three focus groups, with two individual interviews conducted instead of a fourth focus group. Given prior work suggests that 80% of themes are discoverable within 2–3 focus groups, it is likely that the present work captures the most critical findings. However, caution should still be exercised when attempting to generalize to the broader population of grandparents. Second, social desirability bias may have led to participants providing more positive accounts of their caregiving practices. However, in all focus groups, participants reported a range of barriers and shared difficulties in promoting healthy movement behaviors, with some participants openly admitting to engaging in unhealthy practices (such as using electronic devices as “babysitters” and bribing children to engage in physical activity with takeaway food). Third, our sample may reflect a more physically active cohort of grandparents than the average population of Australian grandparents due to their willingness and interest to engage in focus groups on the topic of physical activity. As such, we may have less insight on the barriers and strategies employed by grandparents who are not physically active, or who are not interested in promoting physical activity.

Fourth, women were overrepresented in the sample. Although grandmothers are significantly more likely to engage in grandchild care and do so more frequently than grandfathers [51–54], the low number of men in this study precluded us from exploring any gender differences in perceptions of the barriers and facilitators to increasing physical activity and minimising screen time. Given previous research has suggested grandmothers are more likely than grandfathers to report that their grandchildren engage in unstructured physical activity [30], research exploring gender differences in grandparents’ perceptions is warranted. Finally, only one researcher coded and analyzed the data. Although this is consistent with the assumptions underpinning our reflexive, interpretivist approach to data analysis, some researchers with certain paradigmatic assumptions may challenge this approach [55].

In addition to the directions for future research outlined prior, we propose several further avenues for work in this area. Exploring the experiences of grandparents from culturally and linguistically diverse backgrounds is warranted, especially given Australia’s multicultural society. Exploring the effectiveness of different intervention strategies that target the barriers and facilitators outlined in this study is also recommended. Finally, exploring grandparents’ previous experiences with physical activity and sedentary behavior – with their own grandparents and with their children – may provide insights into how movement behaviors are perceived and transmitted intergenerationally.

## Conclusions

Identifying grandparents’ perceptions of the barriers and enablers to promoting physical activity and reducing screen time in grandchildren is an important step towards optimizing the role grandparents can play in care. The need to develop programs and policies that support grandparents to promote physical activity and reduce screen time is evident. The present study provides several important insights for the development of interventions that aim to support grandparents as they improve their grandchildren’s movement behaviors. Such interventions not only have the potential to increase physical activity and reduce screen time in children; they may also result in improvements to grandparents’ own movement behaviors and health.

## Data Availability

The datasets generated and analysed during the current study are not publicly available but are available from the corresponding author upon reasonable request.
